# Droplets: Unconventional Protocell Model with Life-Like Dynamics and Room to Grow

**DOI:** 10.3390/life4041038

**Published:** 2014-12-17

**Authors:** Martin M Hanczyc

**Affiliations:** Centre for Integrative Biology (CIBIO), University of Trento, Via Sommarive, 9 I-38123 Povo (TN), Italy; E-Mail: martin.hanczyc@unitn.it

**Keywords:** artificial cells, droplets, convection, emergence of life, fluid dynamics, minimal cells, origin of life, protocells

## Abstract

Over the past few decades, several protocell models have been developed that mimic certain essential characteristics of living cells. These protocells tend to be highly reductionist simplifications of living cells with prominent bilayer membrane boundaries, encapsulated metabolisms and/or encapsulated biologically-derived polymers as potential sources of information coding. In parallel with this conventional work, a novel protocell model based on droplets is also being developed. Such water-in-oil and oil-in-water droplet systems can possess chemical and biochemical transformations and biomolecule production, self-movement, self-division, individuality, group dynamics, and perhaps the fundamentals of intelligent systems and evolution. Given the diverse functionality possible with droplets as mimics of living cells, this system has the potential to be the first true embodiment of artificial life that is an orthologous departure from the one familiar type of biological life. This paper will synthesize the recent activity to develop droplets as protocell models.

## 1. Droplets

The droplet consists simply of a liquid compartment that is highly insoluble in another liquid. Typically when the system consists of immiscible fluids, the interfacial tension is high (e.g., nitrobenzene in water at 25 °C is approximately 27 mN/m). If the two phases are at all miscible, the droplet will slowly decrease in volume and dissolve uniformly and predictably following the Epstein-Plesset model [1]. Simply by placing one immiscible liquid into another, a droplet will form and not dissolve. However when surfactants are added, the system can become very dynamic. Surfactants tend to self-assemble at the liquid-liquid interface and mitigate the interfacial tension between the liquids dynamically. When the system is far from equilibrium due to the initial concentration of chemicals in one phase or due to chemical transformation, the distribution of surfactants and therefore the interfacial tension can be non-uniform. Small convective flows develop and can grow [2,3,4]. Under these conditions flow structures form, primarily due to Marangoni-type instabilities [5,6]. These flow fields (both within the droplet and in the liquid proximal to the droplet) can affect the shape, the state of the droplet and its dynamic properties.

Different types of hydrophobic chemicals can be solvated into oil droplets and hydrophilic chemicals into water droplets. Therefore droplets can act as containers. Chemicals can either become stably entrapped in a droplet or diffuse out, depending on their properties. Mass transfer in oil-water-surfactant systems is also possible (e.g., [7]). Oil-in-water and water-in-oil droplet systems, such as presented here, are intended as artificial life models that are able to possess some of the properties of living biological systems. These are examples of bottom-up synthetic biology where the properties of living matter are constructed from “the simpler to the more complex, beginning with the reproduction of the more elementary vital phenomena” [8]. Emulsion droplet systems are unlikely to be the direct progenitors of the first biological cells due to their structure and content alone. However droplet systems provide an experimental framework for synthetic biology that is different from other protocell model systems such as vesicles [9] with distinct advantages. Exploiting the fluid dynamical properties of droplets using different chemistries, this general droplet platform can be custom purposed as described in this review towards creating models for artificial life, targeted applications and exploration of origin of life scenarios not easily done with other supramolecular platforms.

## 2. Individuality

One primary concern in creating artificial systems as protocells is if the created units are completely uniform or variable as in living systems. The encapsulation of an ensemble of molecular types and functions can lead to individual compartments that are not clonal but compositionally and functionally individual. Due to the small internal volume of typical vesicle based or emulsion based containers, a high degree of noise and fluctuation is expected, with certain up concentration mechanisms at play [10]. Stochasticity has been demonstrated in both vesicle systems and droplet emulsion systems where gene expression machinery has been encapsulated [11,12]. Therefore variation, right down to the protocell level [13], can be explained and is expected in such artificial systems. Such stocasticity and variation in individual protocell composition and function can form the basis for selection and evolution in such systems. For example, nitrobenzene droplets seeded with oleic acid then placed in an aqueous environment of oleate micelles will produce self-movement [14]. However nitrobenzene droplets in the same environment but seeded with the cationic amphiphile CTAB (cetyl trimethylammonium bromide) will produce self-division [15]. In addition, Toyota’s group showed that when the length of the linker between two cationic surfactants is varied in a gemini surfactant droplet system, the droplets will either show self-motion or fusion [16]. Therefore, the individual behavior and characteristics of a droplet can be linked to individual droplet content.

## 3. Self-Division and Replication Cycle

**Figure 1:**
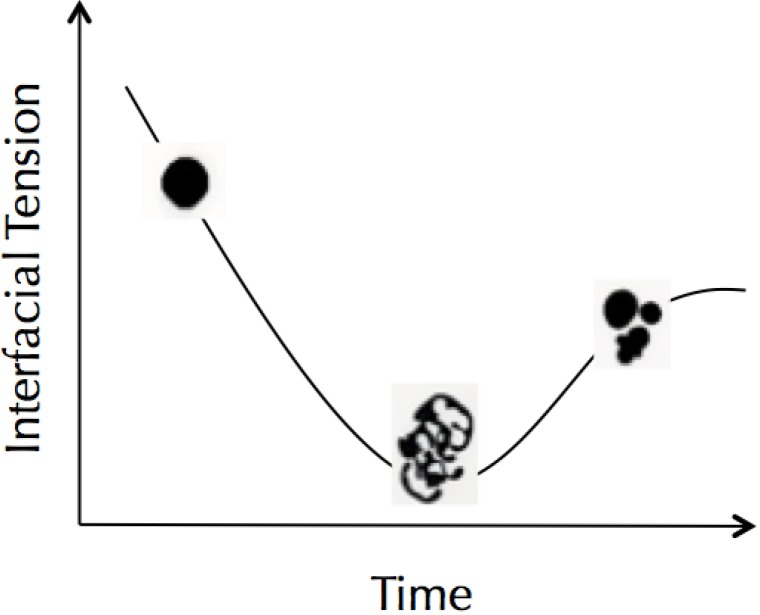
Self-dividing droplet. A representational temporal progression of the droplet transformation from a single droplet to several daughter droplets. As the system approaches equilibrium the droplet initially spherical reaches very low interfacial tension where it can distort and divide. At equilibrium the interfacial tension rises and the daughter droplets round up. For original droplet fission video, see [15].

Fluid dynamics dominate the behavior of chemical droplets. Browne *et al.* [17] showed how organic droplets containing a single surfactant could divide while dissolving as they approach equilibrium, with the extent of division controlled primarily by pH. The macroscale droplets consisting of dichloromethane mixed with the monocarboxylic acid, 2-hexyldecanoic acid, were produced in water at pH 12, and they divided continuously until they reached the nanoscale. In an alternative system, Caschera *et al.* [15] showed how a droplet system composed of two interacting catanionic surfactants can trigger fluid dynamics that pull a droplet apart into daughter droplets. The system starts far from equilibrium with one surfactant in one phase and the oppositely charged surfactant solvated in the other phase. Within seconds after a droplet is formed in the aqueous solution the droplet becomes unstable and the internal flow dynamics promote division. Because of the mixture of catanionic surfactant there is a transient temporal window of very low interfacial tension where flow forces can perturb the droplet. However as the distribution of the surfactants approaches equilibrium, the interfacial tension of the system rises and the droplet scan no longer self-divide or dissolve, see Figure 1. In this general way the singular temporal division event is comparable to cellular division. By coupling droplet self-division with droplet fusion, we were able to demonstrate a recursive droplet fusion-fission cycle [15]. Recently Derényi and Lagzi [18] showed that timing of droplet self-division can be effected through a chemical pH clock reaction (see video [19]).

## 4. Self-Propelled Oil Droplet

Droplets formed in the presence of surfactants can also form organized fluid dynamics and self-motion [20,21,22]. For a recent review of self-moving droplets, as well as vesicles and other particles see [23]. There are three main configurations for self-moving droplets:

(1) no reactive chemistry
(2) onboard fuel
(3) onboard catalyst

The link to the environment is established differently for each configuration and this affects the degree of autonomy and life-time of movement.

For the first type a droplet is placed into an environment that is externally patterned to produce a tension gradient on the droplet. The gradient can be in the chemical composition of the solution [24,25] or on the underlying surface [26,27,28]. The droplet will continue to move as long as the gradient is in place. No chemical reaction is necessary. Rather the droplet will move as long as the distribution of chemicals acts as a gradient that affects the interfacial tension of the droplet or the patterned surface sustains the wettability difference of the droplet with the surface. The movement of the droplet in this case is closest in kind to a ball rolling down a hill. The droplet will not move in an environment where no external gradient is imposed. When a gradient is present, the droplet will move to the lowest energy state of the system and then stop unless another gradient is presented.

**Figure 2:**
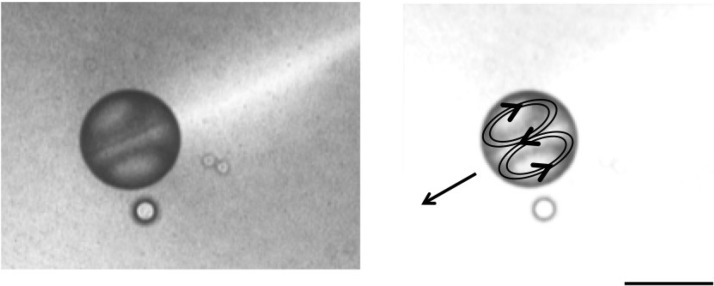
Self-moving droplets consisting of oleic anhydride and nitrobenzene. A micrograph shows the visual pattern in a spherical droplet that is moving by convective flow (left). The same micrograph overlayed with direction of droplet movement as well as the convective flow structures. Size bar: 100 μm.

In the second type, the droplet movement itself creates the local chemical gradient by using onboard fuel [14]. The asymmetry of the droplet is sustained by the chemical reaction and allows for movement for minutes to hours. The droplet follows this self-created chemical gradient and will continue to move until the fuel is exhausted or the waste products build up to a degree that appreciably slows the reaction rate. We showed how a droplet of nitrobenzene that contains oleic anhydride as a source of chemical potential and surfactant can become a self-motile convective droplet, see Figure 2 [14]. Since the droplet system contains the monocarboxylic acid surfactant oleic acid, the system dynamics are sensitive to pH. Therefore droplet follows the pH gradient (internally generated or externally imposed) and is therefore capable of chemotaxis as found previously only in living systems. It is notable that the droplet when moving in such a system has an emergent mechanism of self-movement that allows it to move directionally away from the waste that it produces. The fluid flow dynamics (in this case convective flow) becomes the feedback loop for sustained motion. The overall effect of sustaining the self through movement may also be an important motivator in living systems. We have been primarily investigating how reactive droplets with onboard fuel move through liquid systems. However a reactive droplet can produce self-movement through chemical modification of a surface. Bain [29], dos Santos and Ondarçuhu [30], and Yoshinaga *et al.* [20] have demonstrated fast droplet motion due to a gradient in wettability created dynamically by a chemical reaction between the droplet and the surface.

In the third type of self-moving droplet, an encapsulated catalyst can fuel the movement of the droplet with the fuel source supplied in the external environment [31]. The catalyst in the droplet processes the external fuel source to fuel its movement. If the fuel source is continually supplied (with the removal of waste) the droplet could in principle move indefinitely, as the catalyst localized within the droplet is not consumed.

The potential utility of such self-propelled droplets has been applied in some interesting contexts. The self-moving droplet system with oleic anhydride has been developed into an alginate-based capsule robot where the chemical droplet acts as the motor [32]. The chemotactic property of such droplets have been exploited by Lagzi *et al.* [24] where droplets consisting of dichloromethane and 2-hexyldecanoic acid where able to follow pH gradients and effectively solve a 2D maze. Recently we developed a decanol droplet system that can follow salt gradients with the ability to reverse the direction of movement repeatedly. We used this system to navigate a topologically complex maze, to carry and release a chemically reactive cargo, to select a stronger concentration gradient from two options, and to initiate chemotaxis by an external temperature stimulus [25]. The maze solving droplets represent self-moving droplets of type 1 (with no reactive chemistry). When presented with gradients of varying magnitude, a droplet of this type will follow the steepest gradient reproducibly to arrive at the lowest energy state of the system. We note that some living organisms follow gradients differently. For example slime mold Physarum polycephalum can also solve a maze but it will do so by initially exploring the many different paths. Once the correct path through the maze is established, this one path will then be reinforced preferentially among the other options [33]. The ability of self-propelled droplets to act as sensors for environmental cues, to couple the sensorial information to directional movement and to be able to transport reactive chemical cargo may have applications in medicine, bioremediation and soft body robotics (e.g., [34]).

## 5. Group Dynamics and Higher Order

Through the chemotactic assays, we have shown that a droplet can sense cues from the chemical environment in the form of chemical gradients. But a droplet can also sense other droplets and accordingly change its behavior [31]. We analyzed the movement of single droplets and two droplets as a function of droplet size (volume). We found that in the first 20 min of movement the smaller droplets tended to stay close to each other while moving in the dish [35]. This kind of communication between droplets served to coordinate group dynamics and was highly dependent on both the scale of the system (with larger droplets not coordinating movement) and droplet shape. In addition we note that the variation in a single droplet’s behavior, analyzed as the stop-go interval, displays a power law distribution indicating some bias in behavior due to memory effects in the system [36]. This analysis is useful for determining if these simple droplets either individually or in populations are capable of higher order processes such as decision-making. The individuality of a droplet may be found in chemical composition [37] coupled with internal flow structures. The memory effect we discovered was not necessarily due to the internal structure of the droplet but may be patterned in the immediate external chemical environment. Due to the close coupling between sensing of the environment and movement of the droplet, we have argued that the simple droplet system can form the basis for cognitive systems and smart materials [38].

If we accept that information can be structure, expressed by structures interacting through laws [39], we can show how information can be introduced to the droplet world in a simple programmable way. We have developed a custom ssDNA anchoring system that can be integrated on the surface of droplets. The anchoring system consists of a phospholipid DSPE (1,2-distearoyl-sn-glycero-3-phosphoethanolamine-*N*-[amino(polyethylene glycol)) convalently linked to polyethylene glycol with a terminal biotin. This lipid can be complexed with a streptavidin protein and additional biotinylated ssDNA oligonucleotides. Otherwise generic oil droplets (consisting of diethyl phthalate) are decorated with different ssDNA information acting as barcodes, which can interact specifically with other complimentary ssDNA information on other oil droplets. Several distinct sets of complementary DNAs can be used to distinguish different droplet populations. By mixing together complementary droplet populations, the droplets can assemble together reversibly forming large interlinked emulsions [40]. The nanoscale DNA information follows molecular pairing laws that can organize microscale droplets into interacting macroscale structures programmably and reversibly. Therefore the change of state in droplet structure and organization can be programmed through molecular polymeric information.

## 6. Prebiotic Droplets

**Figure 3:**
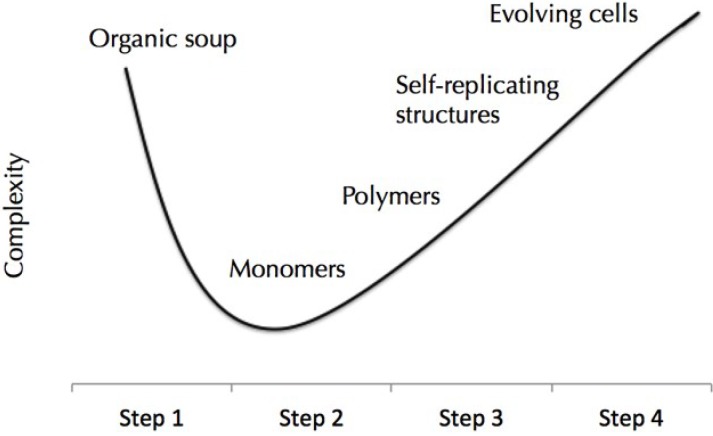
A conceptual model for the origin of life that starts with a high degree of molecular complexity.

A typical chemical droplet experiment involves the careful addition of a few choice and pure chemicals. However it has been argued that pure chemical substances are not realistic components of origin of life protocells. In fact all prebiotic synthesis scenarios produce a highly complex mixture of organic molecules when starting from simple precursors. It is from this huge molecular complexity that life had to organize into protocells and cells, see Figure 3. Mechanisms by which life can emerge from this prebiotic soup are of great interest. To this end we tested a droplet based protocell that used not pure fuel for propulsion such as oleic anhydride [14] but used instead a prebiotic soup based on hydrogen cyanide self-polymerization and hydrolysis. Under these more realistic prebiotic conditions we were able to produce self-motile oil droplets that were also capable of chemotaxis [41]. It is noted that in these experiments the full molecular complexity of a prebiotic soup was added to the droplet system without fractionation or purification. There were likely molecules present in the soup that would both promote and inhibit droplet movement. Nevertheless, the system was able to organize itself into self-propelled droplets that emerged with internal mechanism to avoid equilibrium.

## 7. DIY Protocells

Most of the experiments presented were performed in chemical laboratories with appropriate safety equipment and with chemicals of high purity. However a simple protocell system can also be made DIY using easily available components. One of the first artificial cell models was developed by the zoologist Otto Bütschli and published in 1892 [42]. In this study Bütschli was interested in studying the shape and movement characteristics of protists such as the amoeba. He created an artificial amoeba with pseudopodia that mimicked at least superficially a living protist. He observed that his artificial system captures some of the lively dynamic properties of the natural living system [43].

Such protocells, as created by Bütschli, can be made simply by adding potash to olive oil, an old practice followed to make soap. Potash from which the element potassium takes it name [44] is made by soaking the ashes of burned trees and plants in water. There are many ways to make potash but I have had success with using the ashes from burned hardwoods. I added enough pure spring water to completely cover the ashes in a one liter plastic container and let the mixture soak for one week with daily stirring. The ashes settle to the bottom and a clear slightly yellow liquid is left at the top. The top clear layer can be extracted and passed through a cloth to remove debris. One can use a fresh egg to check if the density of the resulting potash liquid is high enough. An egg that does not sink into the liquid but floats at the top indicates that the leaching of salts from the ashes was successful. When making potash solution one should be careful as the resulting solution (also called lye) is rich in potassium hydroxide, is caustic, and can cause a chemical burn.

**Figure 4:**
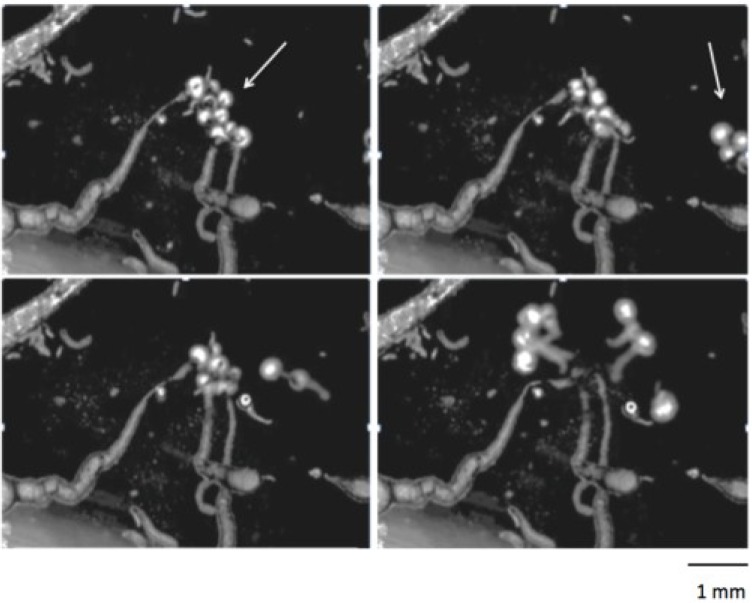
Bütschli aqueous droplets reacting in olive oil. Arrow in first panel shows the initial group of the droplets. Arrow in the next panel shows the invading droplets. Time course: 30, 60, 100, 180 s. Visualized under low magnification with an inverted light microscope, for more technical details see [43]. Size bar 1 mm. For the complete video, see [45].

When a few droplets of the potash solution are added to fresh olive oil, the aqueous droplets react through a saponification reaction and will start to migrate through the oil and eventually break into smaller droplets. One can examine the behavior of these chemical droplets by eye and also with a microscope. An example of microscopic group dynamics of such droplets is shown in Figure 4. Here the aqueous droplets group together while they make long visible trails of sodium-fatty soap precipitate. When a small group of droplets invade and join the larger group, the droplets quickly disperse as if reaching a threshold. Such dynamics are easily observable with this DIY system. If the reaction is slow or terminates quickly, then the oil phase may be too old. For the best lively movement, fresh olive oil must be used. Other oils such as canola with high triglyceride content may also be used. Otherwise if the system is slowly reacting, the potash solution may be too dilute. The solution can be concentrated by carefully evaporating the excess water. To color the aqueous droplets for macroscopic visualization, the juice from blueberries (fresh or frozen) can be added to the potash solution.

## 8. Conclusions

Over the past 130 years or so, several protocell models have been created and developed. Starting with Bütschli’s amoeboid-like droplets in the 1880’s, coacervates, proteinoid microspheres, and vesicles have all been established as simplified experimental models of natural biological cells [9]. Here we focus on chemical droplets as protocell models. When far from equilibrium, these droplets are dynamic and display life-like behaviors. So far we have set the initial conditions of the experiment far from equilibrium and then the system remains closed. In order to further exploit the non-equilibrium dynamics of droplets, we have developed a robotic workstation that not only can perform droplet experiments but monitors the experiments and can modify the course of an experiment in real time. We have already demonstrated that such a robotic platform can maintain the non-equilibrium state of droplets and can effect state change in selected droplets in a population, essentially creating a controlled but more thermodynamically open system [46].

Because chemical droplets are easy to construct, several instances of self-moving, self-dividing, and chemotactic droplets have already been realized. In other self-propelled systems such as the classic camphor in water [47,48], tethered nanospheres [49], molecular motors embedded on surfaces [50], peroxide-activated goldplatinum nanorods [51], Janus particles [52], and catalytic microtubular jet engines [53], it is difficult to imagine how to develop such systems into a customizable, generalizable dynamic technical platforms. Droplets on the other hand are robust, economical and easily customizable. Directed responses to light [54], heat [55,56,57], pH [14,18,24,58], redox-active surfactants and electrochemistry [59], magnetic fields [60], and salt stimuli [25] have been demonstrated.

As protocell models, it is argued that oil droplets present simple self-organized systems that realize emergent movement to avoid equilibrium. This is analogous to the motivation for movement in living biological systems. Although biological systems use much more sophisticated mechanisms for movement such as rotating flagella or actin-myosin polymerized cytoskeleton, the organization of matter into sensing and self-moving matter might be an easily accessible solution for simple prebiotic systems to sustain themselves and avoid equilibrium. With regard to synthesizing life using protocells simply from self-assembly of components, no one has successfully demonstrated a living protocell based on the principle of self-assembly despite more than 100 years of experimentation. This is one of the main motivations for producing a different type of protocell based on self-organizing self-propulsion [61]. Immediately from the first analysis of self-movement and chemotaxis we were able to establish the link between environmental sensing and direction and mode of motion. This intimate link between the environment and protocell behavior may form the basis for higher order functionality in such simple nonliving protocell systems [38]. The idea of dynamic droplets as protocells promotes more open thinking about how non-living matter might self-organize into evolving matter that adapts over time to a changing environment. Perhaps such simple dynamics in simple physical systems will constitute in the future the first true embodiment of artificial life that is an orthologous departure from the one familiar type of biological life based on DNA and protein enzymes. This lifts the constraints of searching for liquid water-based life in the universe where such conditions are not feasible [62].
